# Positive Outcomes of Comprehensive Exercise Program on Restoration of Functional Level and Quality of Life in a Patient With Rheumatoid Arthritis: A Case Report

**DOI:** 10.7759/cureus.30553

**Published:** 2022-10-21

**Authors:** Anushka Raipure, Ruhi Kumbhare, Rashmi R Walke

**Affiliations:** 1 Cardiorespiratory Physiotherapy, Datta Meghe Institute of Medical Sciences, Ravi Nair Physiotherapy College, Wardha, IND

**Keywords:** elderly onset rheumatoid arthritis, pulmonary rehabilitation, rheumatoid arthritis, physical therapy, panchakarma

## Abstract

A relatively recurrent inflammatory disease that is autoimmune, affecting the tissue that lines the joints and tendons, is rheumatoid arthritis (RA). Genome-wide association research has discovered additional genetic markers. The cornerstones of current RA care strategies include anti-inflammatory pharmaceuticals, disease-modifying anti-rheumatic drugs, joint protection and energy conservation, exercise, assistive devices, splinting, orthotics, and surgical treatment. Cardiovascular ailment is still the leading cause of mortality, and those with autoimmune diseases are far more likely to have cardiovascular disease. We present a case of RA with a history of hypothyroidism, hypertension, and diabetes mellitus. She has also taken Ayurveda treatment panchakarma for the same. Physiotherapy interventions included resistance training and aerobic exercises, which showed appreciable results on the Numeric Pain Rating Scale and Multidimensional Health Assessment Questionnaire.

## Introduction

A prevalent chronic systemic inflammatory autoimmune illness that affects the synovial tissue is rheumatoid arthritis (RA) [1]. Diagnosing and treating somatic symptoms, pain, stiffness, and edema is essential since these symptoms frequently arise in clinical practice [2]. The most apparent sign of arthritis is bilateral discomfort and puffiness in the hands, wrists, feet, and knees. The pillars of current RA care strategies include anti-inflammatory medications, disease-modifying anti-rheumatic drugs, joint protection and energy conservation education, exercises, assistive devices, splinting, orthotics, and surgical treatment [3]. RA is also linked to systemic symptoms, most significantly increasing the risk of cardiovascular diseases [4].

Cardiovascular ailment is still the leading cause of mortality, and those with autoimmune diseases are far more likely [5]. Hypertension is a key modifiable risk factor for cardiovascular disease that is also found in patients with autoimmune disorders [6]. The chronic inflammatory load in RA may contribute to increased arterial stiffness, one of the physiologic reasons for high systolic blood pressure (BP), suggesting a possible relationship between inflammation and hypertension in this illness [7].

We present a case of RA with a history of hypothyroidism, hypertension, and diabetes mellitus. She has also taken Ayurveda treatment panchakarma for the same. Physiotherapy interventions included resistance training and aerobic exercises, which showed appreciable results on the Numeric Pain Rating Scale (NPRS) and Multidimensional Health Assessment Questionnaire (MDHAQ). 

The purpose of the case report was to study the effects of pulmonary rehabilitation, aerobic training, strength training, and balance training in a patient with RA having co-morbidities like hypertension, hypothyroidism, and diabetes mellitus.

## Case presentation

A 54-year-old female came to the physiotherapy department complaining of body pain, back pain, and breathlessness. The patient gave a history of progressive deteriorating pain and swelling in the wrist, hand, and legs. The patient also complained of pain in the spine for 3-4 years. She gave a history of hypothyroidism and hypertension for 28 years. Family history was significant for thyroid. The patient had substantial swelling on the wrist, elbow, and ankle. These joints were erythematous and sensitive to the touch. She also gave a history of taking panchakarma treatment six months back for the same and was also advised to follow a diet.

Timeline

The patient was diagnosed with hypertension and hypothyroidism 28 years back. The patient started panchakarma treatment for RA on 12/4/21. Following this, she took physiotherapy treatment on 8/10/2021.

Clinical findings

The therapist obtained the patient's consent before the examination. On observation, swelling and peripheral edema were present. Deformities over joints were not present. On palpation, the local temperature was average. The affected joints showed grade 1 tenderness. There was grade 3 edema and a capillary filling time of more than 2 seconds. 

Diagnostic assessment

Standing dynamic lumbar spine range-of-motion testing on the patient revealed functional limitations in every direction. Lumbar flexion was 0-30 degrees, the lumbar extension was 0-10 degrees, and the side flexion was 0-10 degrees. The patient complains of pain in all degrees of freedom throughout the range of motion. On NPRS, the pain pre-rehabilitation was 9/10. The MDHAQ score on pre-rehabilitation was 1.3 (moderate).

Physiotherapy intervention

Hydrotherapy, electrical stimulation, and hot/cold treatments are some techniques used in physiotherapy. Our rehabilitation programs for RA patients have four main goals: to reduce pain, prevent disability, increase functional capacity, and educate the patient. The therapist implemented the physiotherapy intervention in a phasic manner (Tables 1-3).

**Table 1:** Shows the physiotherapy interventions in phase 1.

Phase 1 (week 1)	Goals	Rationale	Exercises
	Patient education	-	It educated the patient about the disease, its progression, complications, associated illnesses, the importance of physiotherapy, exercise advice, and pain education neuroscience.
	To decrease pain	Hot packs induce relaxation, thereby reducing pain. Electrical stimulation works on the pain gate mechanism, thus relieving pain.	The therapist gave transcutaneous electrical nerve stimulation (TENS) for 10 minutes. Hot packs 15-20 minutes once a day.
	To increase the range of motion	We are maintaining and regaining complete joint movements to facilitate activities of daily living.	With minimal assistance, the patient made moderate, continuous movements. The therapist instructed the patient in isometric exercises. During the training, the therapist supervised the protocol.
	To increase muscular strength	Resistive and repetitive exercises promote the strengthening of the muscles by conditioning them.	The therapist trained the major muscle groups at 40% of one repetition maximum. Hand exercises, wrist exercises, ankle exercises (10 repetitions = 1 set).
	To prevent cardiovascular complications	To enhance endurance and promote compelling cardiovascular well-being.	The patient started low-intensity cardiovascular exercise based on a walking program for 10 minutes five days weekly—step target of 1000 steps.
	To prevent respiratory complications	Breathing exercises for minimizing adhesion formation.	Deep breathing exercises 10 repetitions (one set), active cycle of breathing technique (one set).
	To prevent postural deviations	The therapist used postural correction to prevent deformities.	The therapist informed the patient about the best positions and suggested the patient use the same approach when performing daily tasks.
	To improve balance	To facilitate walking and reduce the risk of falls.	One leg stance (5 seconds hold).

**Table 2:** Depicts the physiotherapy intervention in phase 2.

Phase 2 (weeks 2-4)	Goals	Rationale and exercise	
	To decrease pain	According to phase 1	
	To increase the range of motion		
	To increase muscular strength	Increasing the number of resistive and repetitive exercises promotes muscle strengthening by conditioning them	The therapist trained the major muscle groups at 60% of one repetition maximum (two sets), hand, wrist, and ankle exercises (two sets).
	To prevent cardiovascular complications	According to phase 1	The patient started moderate-intensity cardiovascular exercise based on a walking program for 15 minutes five days weekly—step target of 2000 steps.
	To prevent respiratory complications		Deep breathing exercises with 10 repetitions (two sets) and active cycle of breathing technique.
	To improve balance		One leg stance (10 seconds hold).

**Table 3:** Shows the physiotherapy intervention in phase 3.

Phase 3 (weeks 4-6)	Goals	Intervention
	To decrease pain	According to phase 1.
	To increase the range of motion	
	To increase muscular strength	The therapist trained the major muscle groups at 80% of one repetition maximum (two sets).
	To prevent cardiovascular complications	The patient started moderate-intensity cardiovascular exercise based on a walking program for 30 minutes five days weekly (step target of 3000 steps).
	To prevent respiratory complications	Deep breathing exercises for 10 repetitions (three sets), and active cycle of breathing technique (three sets).
	To improve balance	One leg stance (15 seconds hold).

The home exercise program was started on week 6. This ensures that the patient's recovery is maintained. The patient was handed the printed workout protocol.

Follow-up and outcome measures

After six weeks, the therapist held a follow-up session. On examination, the patient had no complaints. Lumbar flexion was 0-55 degrees, the lumbar extension was 0-230 degrees, and the side flexion was 0-25 degrees. By the end of six weeks, the patient reported a full and relatively less painful range of motion. The patient-reported pain on NPRS was 1/10. MDHAQ score post-rehabilitation was 0.3 (typical) (Table 4).

**Table 4:** Shows the comparison between pre- and post-rehabilitation assessments.

Outcome measures	Pre-rehabilitation	Post-rehabilitation
Numerical Pain Rating Scale	9/10	1/10
Multidimensional Health Assessment Questionnaire (MDHAQ) score post-rehabilitation	1.3	0.3

## Discussion

Women are more likely to be affected by RA, which has an unclear origin. Although it can begin at any age, there is a tendency for symptoms to appear around the age of 40. The most prevalent complaint among patients is pain, characterized by acute polyarthritis, chronic synovitis in the hands, distal joint edema, morning stiffness, and muscle weakness. Additionally, people with RA have reduced oxygen concentrations in their hand and arm muscles, which is consistent with the current study. Soft tissue alterations, tendons degradation, and worsening of deformities can result from these changes [8].

According to a study by Patel et al., panchakarma and manual passive stretching provided good pain and stiffness alleviation for the patient [9]. Ayurveda can manage RA with the food regimen as tips and advice in the prescription of Amavata (accumulation of ama in joints leading to RA) [10]. Concerning a review article by Bullock et al., patients with RA benefit from both physical and occupational therapy. They are encouraged to exercise often to maintain joint mobility and build up the joints' musculature. The patient can reduce exercise-related discomfort by using heat and cold compresses before and after the activity [11].

According to a review article by Hernández-Hernández, patients with RA benefit from aerobic and resistance exercises done 2-3 times a week for 30-60 minutes. The benefits include reduced discomfort, improved muscle function, and a delay in the development of impairments [12]. According to a review article published in 2018, exercises, physical modalities, orthoses, assistive devices, and nutritional therapies are examples of nonpharmacological treatments for RA. Adults with RA should be instructed and encouraged to engage in an efficient exercise program that includes moderate strength training and aerobic activity. Patients with RA should also perform at-home hand exercises [13]. A randomized control trial conducted by Azeez et al. proved that in RA patients, exercise (cardiovascular and strength training) has a significant and positive impact on cognitive and physical function. Physical activity is, therefore, beneficial and safe for treating long-term RA and is an essential component of the patient's overall therapy [14].

Physiotherapy is a helpful tool used in all stages of the disease to improve joint mobility, muscle strength, coordination, flexibility, and aerobic capacity, thereby preserving and restoring the patient's general functional ability. People with chronic inflammatory arthritis frequently have hypotrophy and muscle weakness due to decreased physical capacity and the continuous consumption of glucocorticoids. Physical activity in the everyday life of the RA patient allows them to stay as functioning as possible within the conditions imposed [15].

Twenty women with RA who had deformities underwent a regimen of exercises designed to strengthen muscles. Randomly chosen subjects were assigned to the study of Rastogi et al. The experimental group participated in 20 physiotherapy sessions that included muscle-strengthening exercises, resistive band exercises, post-isometric relaxation, joint mobilization, neuromuscular electrical stimulation, Kaltenborn joint mobilization, art-based intervention like Origami and paper quelling, conditioning exercise program, and laser therapy. The experimental group's subjects' functionality and muscle strength dramatically increased after physiotherapy sessions [16].

For physical activity to have positive clinical effects and not cause pain, it should last for at least 20 minutes, twice a week, with an increase in 60% of the predicted heart rate for the age, according to Cooney et al. Dynamic exercise considerably improves the quality of life of people with RA compared to the traditional joint rehabilitation program. This study employed a rehabilitation program through physical activity and showed positive benefits on patients' pain and quality of life, notably in the pain domains and vitality [17].

## Conclusions

In this study, physical therapy, with an emphasis on manual treatment and therapeutic exercise, was used to treat a case of RA. The exercise protocol was according to the American College of Sports Medicine guidelines and the FITT (frequency, intensity, time, and type) principle, which gave noticeable positive results post-treatment. This case study also emphasizes the use of panchakarma to help relieve the symptoms, along with an intensive physiotherapy program.
